# Panoramic hyperspectral optical mapping of cardiac membrane potential and tissue type

**DOI:** 10.1117/1.JBO.31.7.076004

**Published:** 2026-07-22

**Authors:** Grant Kowalik, Rebekah Russo, Murray Loew, David Mendelowitz, Emilia Entcheva, Matthew W. Kay

**Affiliations:** a The George Washington University, School of Engineering and Applied Science, Department of Biomedical Engineering, Washington, District of Columbia, United States; b The George Washington University, School of Medicine and Health Sciences, Department of Pharmacology & Physiology, Washington, District of Columbia, United States

**Keywords:** myocardial infarction, panoramic, hyperspectral imaging, optical mapping, cardiac arrhythmia

## Abstract

**Significance:**

Cardiac panoramic optical mapping is a powerful approach for studying action potential dispersion and mapping arrhythmia triggers and propagation pathways over the entire surface of the heart. However, tissue type (muscle, connective tissue, and infarct scar) is also important for interpreting mapping data and is difficult to identify using optical mapping data alone.

**Aim:**

Panoramically map transmembrane potential and tissue type from the surface of infarcted hearts for correlative analysis of cardiac structure and function.

**Approach:**

We developed a multimodal panoramic imaging system to map epicardial tissue type (determined by collagen content) using a line-scan hyperspectral camera and a precision stage to translate and rotate the heart while illuminating the epicardial surface with UV light. Transmembrane potential was subsequently optically mapped by imaging a potentiometric probe with four high speed CMOS cameras position around the heart. The epicardial surface was reconstructed for each heart using images acquired every 3.6 deg of rotation, onto which hyperspectral and optical mapping data were texture mapped. All cameras were registered to one coordinate frame using a calibration procedure.

**Results:**

This system combines, for the first time, high-resolution hyperspectral imaging with optical mapping for quantitative correlative tissue structure-function analyses. It was used to study excitation wave propagation and action potentials across the surface of perfused rat hearts having a four-week-old infarct. The spectral band of collagen fluorescence (400 to 520 nm) revealed infarcted and border zone tissue. PVCs and reentrant activity were observed in 3 of 4 hearts at S1-S2 pacing intervals between 80 and 65 msec (S1 = 150 msec). PVCs originated near the infarct border and propagated around the infarct. Using the integral of spectral intensity from 400 to 435 nm, a k-means clustering algorithm classified each mapped site as either healthy, border zone, or infarcted tissue. Average action potential duration within those tissue types was longest for infarcted tissue, shorter for border zone tissue, and shortest for healthy tissue, a preliminary result that is consistent with the effect of an infarct on ventricular electrophysiology.

**Conclusions:**

This work demonstrates that panoramic hyperspectral mapping of tissue type and transmembrane potential is a powerful approach that enables functional mapping data to be analyzed within the context of local tissue type (healthy, infarct, and border) in living hearts.

## 1 Introduction

Electrical activity from the human heart was first recorded in 1887, more than 135 years ago.^1^ The electrocardiogram (ECG), as a noninvasive signal from the body surface, became an indispensable clinical tool in the early 1900s. After over a century of use, a deep understanding of the minute morphological features of the ECG was developed and linked to deviations in cellular level bioelectric signals at specific locations. Yet, cardiac arrhythmias are complex space-time phenomena that cannot be reconstructed fully from the ECG and require more detailed mapping. In 1915, Lewis and Rothschild^2^ positioned multiple electrodes on the surface of canine hearts to meticulously examine how cardiac excitation waves travel within different regions (ventricles, atria, and Purkinje fibers), providing an appreciation of how structure influences wave propagation. Later, Durrer’s detailed electrical activation maps of the human heart,^3^ also based on multiple electrode recordings, entered medical textbooks.

An important technological development, known as cardiac optical mapping, emerged in the late 1970s^4^^–^^6^ wherein fluorescent probes are used to visualize fast changes in cardiomyocyte membrane potential. Cardiac optical mapping soon provided spatial resolutions that surpassed even the densest mapping electrode arrays.^7^ Like electrode arrays, local conduction velocities can be measured using optical mapping; yet, importantly, optical mapping also provides reliable information regarding myocyte excitation and repolarization (action potentials) at multiple locations and enables excitation wave trajectories and dynamics to be studied in exquisite detail in a contact-free manner.^8^^–^^10^ By the late 1990s, optical mapping became instrumental in testing new biophysical theories of the origin and evolution of complex life-threatening arrhythmias in living hearts,^7^^,^^11^^–^^13^ enabling measurements that were not possible with electrode arrays. Reentrant waves as drivers of atrial and ventricular arrhythmias were visualized optically in great detail for the first time.^11^^,^^13^^,^^14^ The complex dynamics of cardiac excitation waves also revealed several important optical mapping limitations. Despite its unmatched space-time resolution, optical mapping in conventional implementations is only a two-dimensional single-view recording of electrical activity within a complex three-dimensional organ. New technologies that optically image the entire surface of the heart from multiple viewpoints were needed for deeper mechanistic studies of arrhythmia initiation and maintenance, which led to the development of panoramic optical mapping systems.

**Fig. 1:**
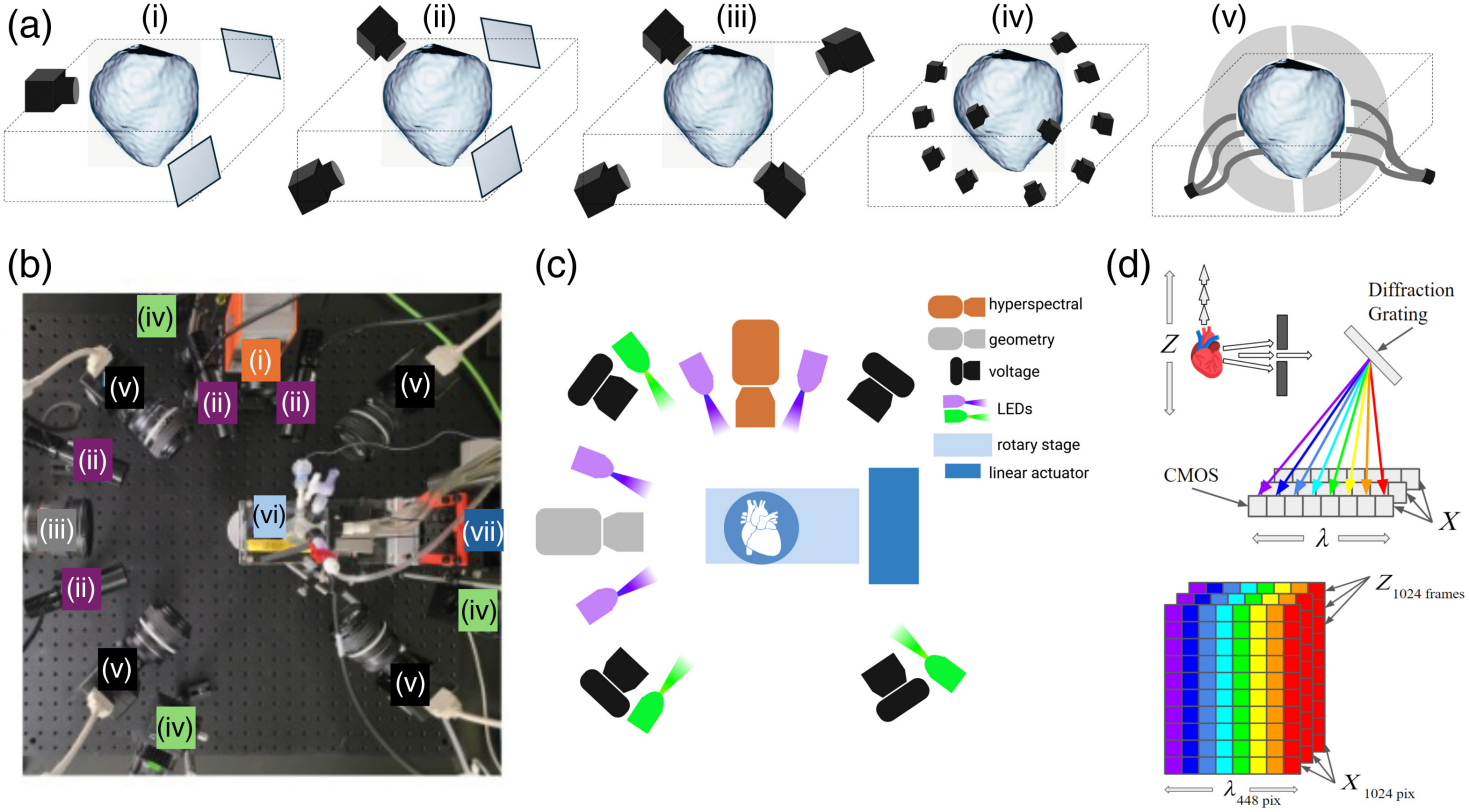
Prior panoramic optical mapping configurations and the configuration of the present system that combines HSI and optical mapping. (a) Several prior imaging configurations for panoramic optical mapping of perfused hearts since 2000, illustrating the use of cameras and mirrors. The image of the heart is modified from Ref. 15. (b) Top view of the current panoramic imaging system showing the positions of each of the 6 cameras, 7 lights, linear actuator and rotary stage used to position the heart: (i) hyperspectral camera, (ii) UV LED spotlights, (iii) geometry camera, (iv) 520 nm LED spotlights, (v) voltage mapping cameras, (vi) rotary stage, (vii) linear actuator. (c) Diagram showing the layout of the system, with an approximate footprint of $2.5\;{\mathrm{ft}}^{2}$. (d) Hyperspectral data cubes (X, Z, λ) composed of concatenated line scans (X, λ) acquired using a Specim FX10e. The acquisition of one line scan is depicted. Light emitted from the heart passes through a slit (42  μm wide and 10.2 mm long), reflects off a diffraction grating, and the separated wavelengths along a line are projected onto a CMOS array (X, 1024 pixels and λ, 448 wavelengths) and imaged. Line scans were concatenated to construct data cubes (X, Z, λ), bottom. Z: vertical position of each line scan (1024 total) from base to apex.

The fundamental elements of panoramic optical mapping, an approach that provides a nearly complete recording of electrical activity from the entire surface of the heart, were developed over the last 25 years [Fig. 1(a)]. In 2000, Bray et al.^16^ published the first prototype of a panoramic optical mapping system that used one high-speed digital camera and mirrors to simultaneously image the posterior and anterior surfaces of the heart.^17^ That system incorporated geometric reconstruction of the epicardial surface onto which electrical recordings were texture mapped [Fig. 1(a)(i)]. In 2004, Kay et al.^15^ improved upon the method by adding a dedicated geometry scanning camera that rotated around the heart, updated surface reconstruction and texture mapping algorithms, and a second high speed digital camera with mirrors [Fig. 1(a)(ii)]. The system was used to panoramically map ventricular fibrillation in perfused swine hearts. The mirrors were soon replaced with two more high speed digital cameras in addition to a rotating geometry scanning camera^18^ [Fig. 1(a)(iii)]. Excitation waves within the panoramic datasets were tracked across the entire epicardial surface and analyzed using graph theory,^10^ revealing important and surprising insights into the short lifetime of rotors during ventricular fibrillation.^18^^–^^20^ Other panoramic optical mapping studies identified proarrhythmic sites of origin in swine hearts^21^ and after myocardial infarction in rabbit hearts.^22^ Continued development of panoramic mapping optimized configurations to enable panoramic mapping of rodent (mouse) hearts,^23^^,^^24^ which is nontrivial due to the need to position many optical components together to image a very small object. Christoph et al. developed the first panoramic optical mapping system for contracting hearts, using computational tools to disentangle the electrical and mechanical signals.^25^ Recent developments take advantage of low-cost LED spotlights and low-cost machine vision cameras^26^ to extend the observation sites (number of cameras) to over a dozen,^27^ providing impressive panoramic spatiotemporal fidelity [Fig. 1(a)(iv)]. A distinctly different approach to panoramic optical imaging and optical stimulation of perfused mouse hearts has also been reported, with some sacrifice in spatial resolution, using fiber optic light guides embedded within a custom enclosure fitted around the heart^28^ [Fig. 1(a)(v)].

In parallel with cardiac optical mapping developments, there has been a long-standing interest in imaging other aspects of cardiac tissue, particularly metabolic and structural properties, because these are often associated with arrhythmogenesis.^29^^–^^31^ Geometric and structural remodeling, including fibrosis, are considered such potent drivers of arrhythmias that they are a central component of current clinical research to develop “digital twins” for cardiac disease patients.^32^^,^^33^ Illuminating cardiac tissue with light at specific wavelengths excites endogenous fluorophores to provide optical assessments of local tissue properties. For example, collagen, elastin, and NADH are excited with UV light. Imaging the resulting fluorescence at longer wavelengths reveals locations that are hypoxic (high NADH fluorescence) or contain dense fibrotic tissue (high collagen and elastin fluorescence) or both. Spectroscopy and optical imaging have been used by our group and others to record changes in tissue fluorescence and absorbance to study oxygen supply and demand in working excised perfused hearts.^31^^,^^34^^–^^36^ Recently, hyperspectral imaging (HSI) has been used to map the spectral tissue characteristics of ablation lesions.^37^^–^^41^ When combined with machine learning methods^42^ and clinically-deployable catheters,^43^ HSI has the potential to improve patient-specific interventions by revealing borders between ablated and nonablated tissue for lesions created during radiofrequency, cryo-, or pulse-field (PFA) ablation procedures. Combining HSI with cardiac panoramic optical mapping could be a powerful new approach to studying arrhythmia mechanisms by co-locating quantitative assessments of tissue structure with electrical activity across the surface of the heart.

To our knowledge, no prior work has described a panoramic optical mapping system for imaging of electrical activity and co-registered panoramic hyperspectral mapping of tissue structure in living perfused hearts. The goal of this study was to develop such a system and to demonstrate its use by optically mapping perfused rat hearts having a 4-week-old infarct.

## 2 Methods

We built a multimodal cardiac panoramic imaging system to map epicardial tissue type using a line-scan hyperspectral camera and transmembrane potential using a potentiometric probe and four high-speed CMOS cameras. Excitation wave propagation and action potential durations (APDs) across the surface of perfused rat hearts having a four-week-old infarct were analyzed from the perspective of local tissue type. The methods and approach to the rat infarct model, heart perfusion, panoramic system design and testing, and data processing and analysis are discussed below.

### 2.1 Rat Model of Myocardial Infarction and Study Protocol

#### 2.1.1 Surgical induction of myocardial infarction

Male Sprague Dawley rats (n=4) were housed at the George Washington University’s Animal Research Facility and fed standard chow ad libitum with free access to water in single housing with a twelve-hour light-dark cycle. After 10 weeks of age, a left ventricular (LV) myocardial infarction (MI) was surgically induced by permanent ligation of the left anterior descending (LAD) coronary artery. Surgical procedure: After anesthetization to a deep surgical plane with isoflurane, ECG leads were attached to the limbs, and incisions were made through the skin and musculature to reveal the ribcage. Another incision was made through the fourth intercostal space, and a rib spreader was inserted. The lungs were retracted, the pericardium was opened, the junction between the pulmonary artery and left atrium was identified, and a ligation 4 mm distal from the left atrium was applied using 6 to 0 taper point silk suture. LAD occlusion was confirmed upon observing a well-defined area of epicardial cyanosis with regional hypokinesia and ECG changes. The incision was closed. Rats were monitored until spontaneous breathing returned, and the intubation tube was removed. Buprenorphine and meloxicam were administered to the surgical site to manage pain and inflammation. Once ambulatory, rats were returned to their cage. Rats were monitored for 3 days post-op, or further if needed, and given additional doses of meloxicam. All animal procedures were approved by the George Washington University’s Institutional Animal Care and Use Committee.

#### 2.1.2 Experimental protocol

**Fig. 2:**
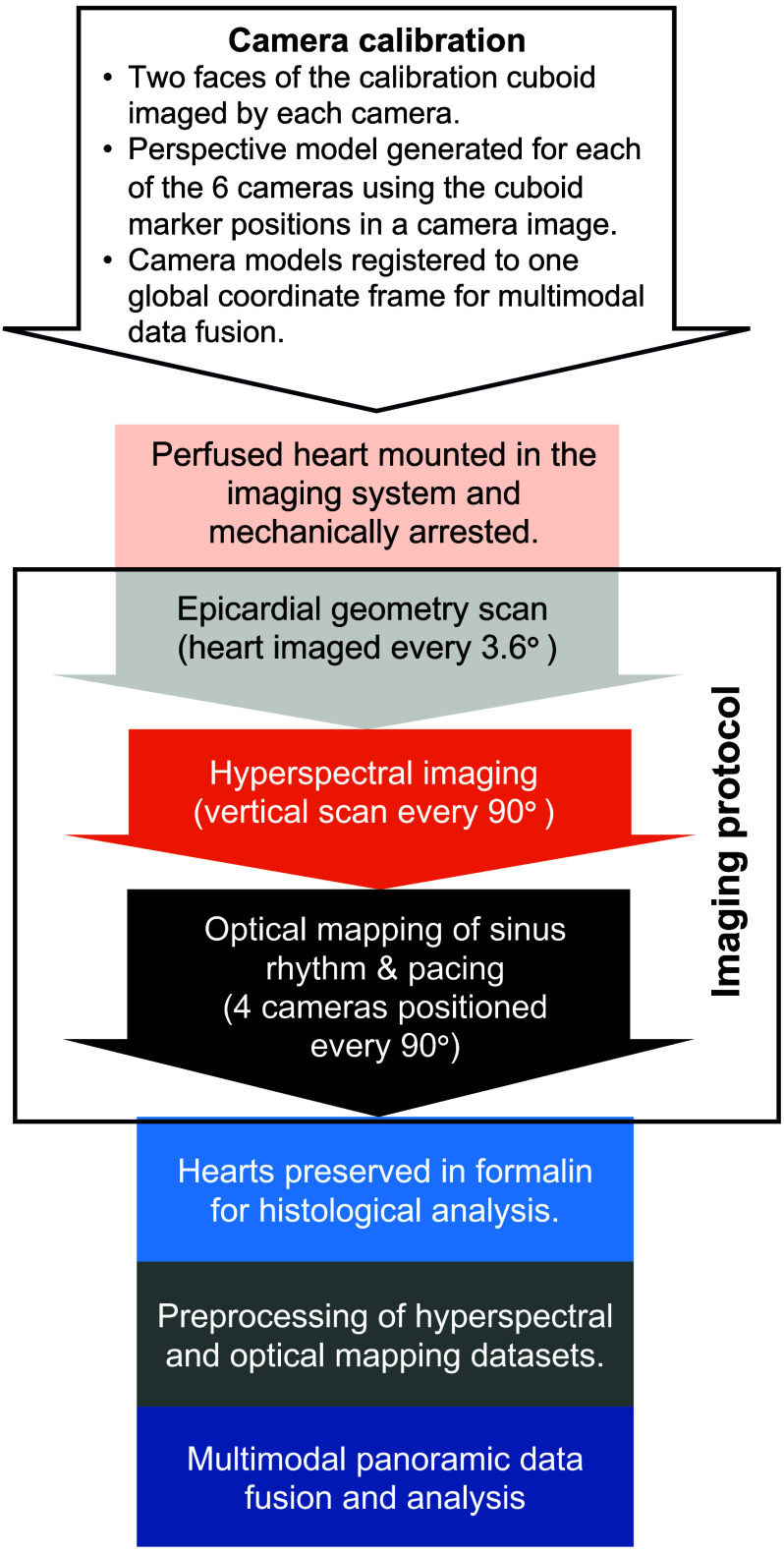
Panoramic mapping workflow for each experiment. The general experimental protocol consisted of calibrating the cameras, excising and perfusing a heart, a multimodal imaging protocol, heart preservation at the end of the experiment, and offline data fusion into panoramic datasets and data analysis.

Hearts were rapidly excised and retrograde perfused for panoramic optical mapping four weeks after MI surgery. After rats were anesthetized (isoflurane) to a deep surgical plane, the heart was excised via a thoracotomy and placed in cold media. The aorta was cannulated, and the vasculature was flushed of blood. The heart was then positioned in the center of the imaging system [Figs. 1(b) and 1(c)] and perfused at constant hydrostatic pressure (75 mmHg) with modified Krebs-Henseleit perfusate bubbled with 95% $O_{2}$, 5% ${\mathrm{CO}}_{2}$ and maintained at 37±0.5°C.^44^ After a 10 min stabilization period, the myosin ATPase inhibitor blebbistatin (Apex Bio) was added to the perfusate (12  μM) to mechanically arrest the heart to prevent motion artifacts during imaging. The imaging protocol (Fig. 2) began after the heart was completely arrested, typically within 5 min. Hearts were first imaged while rotated to reconstruct the epicardial surface geometry, then hyperspectrally imaged [Fig. 1(d)) to panoramically map tissue type. A bolus dose of RH237 (Invitrogen) was injected into aortic flow (final concentration of 1.34  μM) to optically map transmembrane potential during sinus rhythm and epicardial pacing.^44^^,^^45^ Hearts were paced (4.0 mA, 2 ms pulse duration) from an electrode placed at the base of the LV. Optical action potentials (APs) were panoramically mapped during decremental pacing (10 msec decrements, 20 pulses each) for cycle lengths from 200 to 90 ms. S1-S2 pacing was then used to induce an arrhythmia where 10 S1 pulses (150 ms cycle length) were followed by a single premature S2 pulse delivered from a second electrode placed on the right ventricle.

#### 2.1.3 Histology

Hearts were preserved in 10% formalin after each study. The LV was sectioned with a transverse cut through the middle of the infarct; the lower half was embedded in paraffin, and 7  μm thick sections of the LV infarct were mounted on slides that were then stained with Masson trichrome to identify muscle connective tissue and nuclei and stained with Movat Pentachrome to identify elastin and collagen content.

### 2.2 Imaging System Components

#### 2.2.1 Cameras and excitation light

The system consists of six cameras (Table 1) and seven LED spotlights [Figs. 1(b) and 1(c)]. Four high speed CMOS cameras (MiCAM Ultima-L, Costa Mesa, California, United States) were each fitted with 50 mm Nikon Nikkor (f1.2) lens and a 655 long pass emission filter (Chroma ET655lp). The geometry camera (Andor IKon-M) was fitted with an 85 mm Samyang (f1.4) lens and a 405 bandpass emission filter (Chroma ET 405/40, Taoyuan City, Taiwan.). The hyperspectral line scan camera (Specim FX10e) had a spectral resolution of 5.5 nm (FWHM) and was fitted with a 10 mm extension tube and a 45 mm Navitar lens. During epicardial geometry scans and HSI, the epicardial surface was illuminated using four 365±5.5  nm LED spotlights (Mightex, Pleasanton, California, United States), with two spotlights positioned on each side of the geometry and hyperspectral camera [Fig. 1(c)]. During panoramic optical mapping, the potentiometric probe RH237 was energized by illuminating the entire epicardial surface with 520±6  nm light from three LED spotlights (Prizmatix) positioned around the heart [Fig. 1(c)].

**Table 1:** Imaging modes and cameras.

Imaging mode	Quantity imaged	Panoramic measurement	Camera model	Lens	Working distance (cm)	# of cameras	# of views	Pixels per image	Image resolution (mm/pix)	Frame rate	Integration time (msec)	Excitation λ (nm)	Emission λ (nm)
Epicardial geometry	Tissue fluorescence	3D epicardial mesh & collagen content	Andor Ikon-M	85 mm Samyang	22	1	100	1024 × 1024	0.028	1 fps	500	365+/−5.5	405+/−20
Hyperspectral (HSI)	Fluorescence spectra	Tissue type	Specim FX10e	45 mm Navitar	15	1	4	1024 × 1024	0.031	50 msec per line	15	365+/−5.5	400 to 1000
Optical mapping	RH237 fluorescence	Optical action potentials	MiCAM Ultima-L	50 mm Nikon	15	4	4	100 × 100	0.244	1000 fps	0.8	520+/−6.0	665 Longpass

#### 2.2.2 Rotation and translation of perfused hearts

Hearts were retrograde perfused in the center of the imaging system by connecting the aortic cannula to a rotating luer lock connector that was attached to the perfusion tubing and held in the center of a rotary stage [Fig. 1(b)]. The rotary stage (Newport URS50CPP) rotated the cannula 360 deg to provide 100 views of the heart that were imaged by the geometry camera to generate a triangular mesh of the epicardial surface. Hearts were then rotated at 90 deg increments to provide four orthogonal views for HSI. The rotary stage was mounted on an 8 mm lead screw linear actuator that moved the heart vertically as the hyperspectral line scan camera scanned the heart every 0.03 mm to generate hyperspectral data cubes [x=1024, z=1024, λ=448, Fig. 1(d)]. After each line scan (50 ms integration time) the heart was raised to the next step. After a full vertical scan, the heart was returned to the original z-position and rotated 90 deg and imaged again. The stepper motor drivers (Stepperonline DM320T, Nanjing, China) of the rotary stage and lead screw were controlled by custom software running on an Arduino Uno, which also triggered the cameras and UV spotlights during epicardial geometry and hyperspectral imaging.

### 2.3 Panoramic Imaging Workflow

The typical workflow for each experiment is illustrated in Fig. 2.

#### 2.3.1 Calibration and registration of cameras to one coordinate frame

**Fig. 3:**
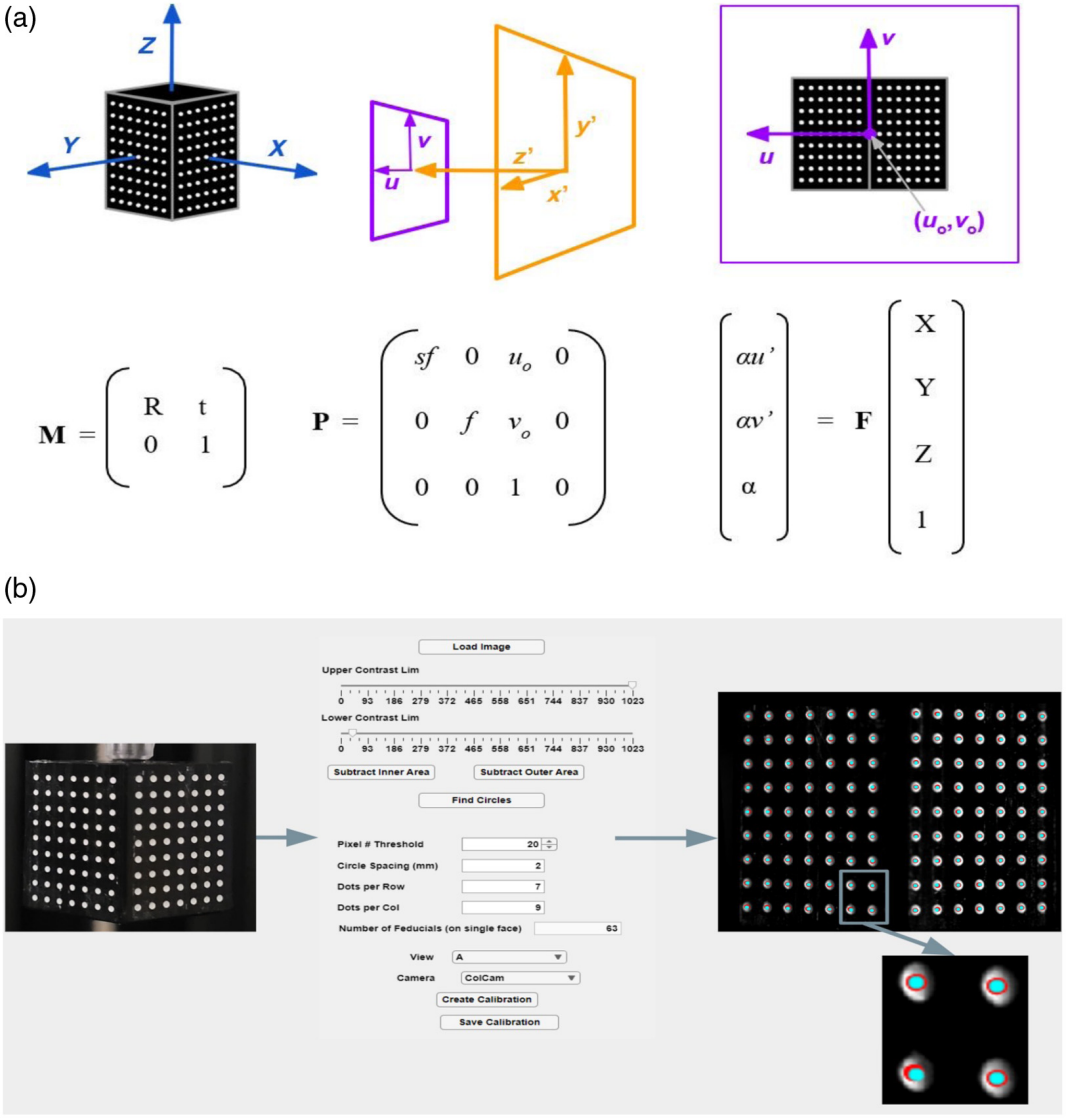
Calibration and generation of a perspective transformation for each camera. (a) Coordinate frames and perspective transformation equations are used to register image data from each camera to a common 3D world frame. Top: the origin of the world frame (X,Y,Z: blue axis) is located at the center of the calibration cuboid. The image frame (purple) defines the axis (u,v) of the 2D projection of a 3D object located in the world frame. The principal point ($u_{0},v_{0}$) is determined by the orientation of the camera in the world frame, which ideally would be at the center of the image. The view frame ($x^{\prime },y^{\prime },z^{\prime }$: yellow axis) defines the 3D origin and view orientation of a pinhole camera model within the world frame. Bottom: pinhole camera perspective transformation equations for registering image pixels to 3D locations within the world frame.^15^^,^^46^ M is a matrix that translates positions in the world frame to the view frame. P projects locations in the view frame onto the image frame, s and f are the camera’s aspect ratio and focal length. F=PM is the pinhole camera perspective transformation that provides $u^{\prime }$ and $v^{\prime }$, which are then used to compute u and v using the camera’s distortion characteristics.^47^ α is a scale factor determined by the camera calibration procedure. (b) Left: typical calibration image acquired by one camera viewpoint showing an edge of the cuboid and two faces. Middle: a screenshot of the custom MATLAB GUI that provides user-selectable contrast thresholds to identify the calibration points, selection of a masking procedure, and selection of the camera view calibration image to analyze. The geometry of the cuboid, the spacing of the calibration points, and the parameters of the perspective camera model are also entered. Right: example of a processed calibration image for one camera view. White regions are the calibration points. Solid red circles are the centroids of the calibration points identified by the calibration algorithm. Solid cyan circles are the calculated back projection of the ideal 3D coordinates of each calibration point.

All cameras (Fig. 1 & Table 1) were registered to one global coordinate frame to accurately co-locate fluorescence spectra and optically mapped transmembrane potential within each element of the epicardial surface mesh. This was done by placing a rectangular cuboid (20×16×16  mm) in the same position as the hearts and imaging it with each camera to generate a calibration image (Fig. 3). The origin of the global coordinate frame was at the center of the cuboid [Fig. 3(a)]. The cuboid was machined from a black polymer material and had a cylindrical nub at the top to attach it to the rotating luer lock connector in the center of the rotary stage. Each cuboid face (20×16  mm) had a 7×9 array of calibration markers: 0.75 mm diameter holes drilled to a depth of 1.25 mm with 2 mm separation. The markers were filled with titanium white acrylic paint for high optical contrast to enable reliable automated detection of the holes [Fig. 3(b)]. A calibration algorithm iteratively solved for the parameters of a pinhole camera perspective transformation [P, Fig. 3(a)] for each camera using the cuboid marker locations in the calibration image (u,v) and world frame (X, Y, Z).^15^^,^^47^ Marker locations in calibration images were identified using a custom Matlab program that located the centroids of the markers with minimal user intervention [Fig. 3(b)].

#### 2.3.2 Epicardial geometry imaging

After positioning in the imaging system (Fig. 2), perfused hearts were illuminated with UV light (365±5.5  nm) to excite endogenous fluorophores (Table 2), rotated using 3.6 deg steps, and imaged at each step with the geometry camera. Silhouettes of the heart were extracted from the image at each step and used to generate a 3D triangular mesh of the epicardial surface, as described below.^15^

**Table 2:** Endogenous fluorophores excitable with UV light.

	Excitation λ (nm)	Emission λ (nm)	Relative fluorescence within normoxic infarcted adult rat myocardium
collagen	310 to 365	370 to 435	High
elastin	315 to 380	375 to 460	Low to moderate
NADH	320 to 370	425 to 510	Low during normoxia
lipofuscin	340 to 395	430 to 460	Low
ceroid	340 to 395	430 to 460	Low
vitamins: K, D, B6	320 to 390	385 to 480	Low

#### 2.3.3 Panoramic hyperspectral imaging (HSI)

Hearts were illuminated with UV light (365±5.5  nm) and imaged using a line scan hyperspectral camera Specim FX10e. The spectral camera was calibrated by the manufacturer to correct for the quantum efficiency of the CMOS chip to provide a linear response based on wavelength, in addition to correcting for the keystone aberration (misregistration of spectral bands) and smile aberration (location-dependent shift in wavelength). Hyperspectral images were generated by raising the heart as the camera acquired line scans (1024 horizontal positions) at 1024 vertical positions from base to apex. The heart was then lowered, rotated 90 deg, and scanned again [Fig. 1(d)]. This was repeated four times, requiring a combined time of 4 min. The acquired spectra at each position (1024×1024) of the hyperspectral images can be used to identify molecular signatures within the tissue (Table 2) to classify tissue type.^48^^–^^50^ Panoramically mapped fluorescence spectra were used to classify epicardial tissue as healthy, infarcted, or peri-infarcted (border zone) using the spectral band that corresponds to collagen fluorescence.

#### 2.3.4 Panoramic optical mapping of transmembrane potential

Green light (520±6  nm) from three high-power LEDs illuminated the entire epicardial surface to excite RH237 for optical mapping of action potentials. RH237 fluorescence was long pass filtered (655 nm) and imaged at 1000  frames/ sec using four high-speed CMOS cameras (100×100  pix). Sinus beats, paced beats, and arrhythmic activity were optically mapped.

### 2.4 Multimodal Panoramic Data Fusion and Analysis

#### 2.4.1 Epicardial surface reconstruction

The perspective transformation (F=PM) for the geometry camera [Fig. 3(a)] and the heart silhouettes extracted from each geometry image were used to generate a 3D triangular mesh of the epicardial surface, as previously described.^15^ An adaptive octree algorithm, in conjunction with an occluding contours algorithm, efficiently identified the volume bounded by the epicardial surface. Delaunay triangulation and an isosurface algorithm generated the final surface mesh onto which panoramic measurements (Table 1) were texture mapped.

#### 2.4.2 Spectral data processing

Raw HSI data (x, z, λ) were spatially filtered (5×5 cone filter) at each wavelength to reduce noise. A custom MATLAB application performed contrast enhancement, removed specular reflection, and enabled viewing of user-defined spectral bands. Spectral characteristics were computed and then normalized within each panoramic dataset to generate data that could be texture mapped onto the epicardial mesh to reveal areas of healthy, border zone, and infarcted tissue. Those characteristics were: (a) The spectral integral from 400 to 435 nm. This spectral band includes the fluorescence of both collagen and elastin (Table 2); however, a histological analysis of the infarcts indicated that the infarcts contain much more collagen than elastin. (b) The spectral angle similarity to a reference spectrum, known as spectral angle mapping [SAM, Eq. (1)]. SAM is similar to a Pearson’s correlation coefficient,^51^ and is useful in identifying spectra with similar morphologies, although it disregards intensity. (c) The spectral similarity to a reference spectrum, known as the normalized spectral similarity score [NS3, Eq. (3)], which uses the SAM [Eq. (1)] in addition to the Euclidean distance between the spectra [Eq. (2)].^52^

$$\alpha =\cos^{-1}(\frac{\overset{C}{\underset{i=1}{\sum }}t_{i}r_{i}}{\sqrt{\overset{C}{\underset{i=1}{\sum }}t_{i}^{2}}\sqrt{\overset{C}{\underset{i=1}{\sum }}r_{i}^{2}}}).$$ (1)

Equation (1): spectral angle mapping (SAM, α). r = reference spectrum. t = sample spectrum. i = wavelength bin number. C = maximum number of bins.

$$A_{\text{Euclidean}}=\sqrt{\frac{1}{C}\overset{C}{\underset{i=1}{\sum }}(t_{i}-r_{i}{)}^{2}}.$$ (2)

Equation (2): Euclidean distance between spectra ($A_{\text{Euclidean}}$). r = reference spectrum. t = sample spectrum. i = wavelength bin number. C = maximum number of bins.

$$\mathrm{NS}3=\sqrt{A_{\mathrm{Euclidean}}^{2}+{\left(1-\;\cos\;(\alpha )\right)}^{2}}.$$ (3)

Equation (3): Normalized spectral similarity score (NS3). $A_{\text{Euclidean}}$ = Euclidean distance between spectra [Eq. (2)]. α= SAM [Eq. (1)].

#### 2.4.3 Optical mapping data processing

Images of RH237 fluorescence acquired at each of the four viewpoints were preprocessed before texture mapping and AP analysis. Saturated pixels and pixels with low fluorescence signal variance were excluded. Signals at each pixel were then normalized between zero and one after correction for baseline drift by subtracting the linear baseline trend at each pixel. Contractile motion tended to recur later in the experiment and was usually caused by atrial contractions, so all RH237 data were further preprocessed to remove motion artifacts at each pixel using an image registration algorithm similar to that of Lebert et al.^53^ An image of the temporal average of each pixel in each RH237 dataset was then computed and subtracted from every image in the dataset. Activation times and APDs were then measured from normalized optical APs at each pixel, as previously described.^44^

#### 2.4.4 Texture mapping

**Fig. 4:**
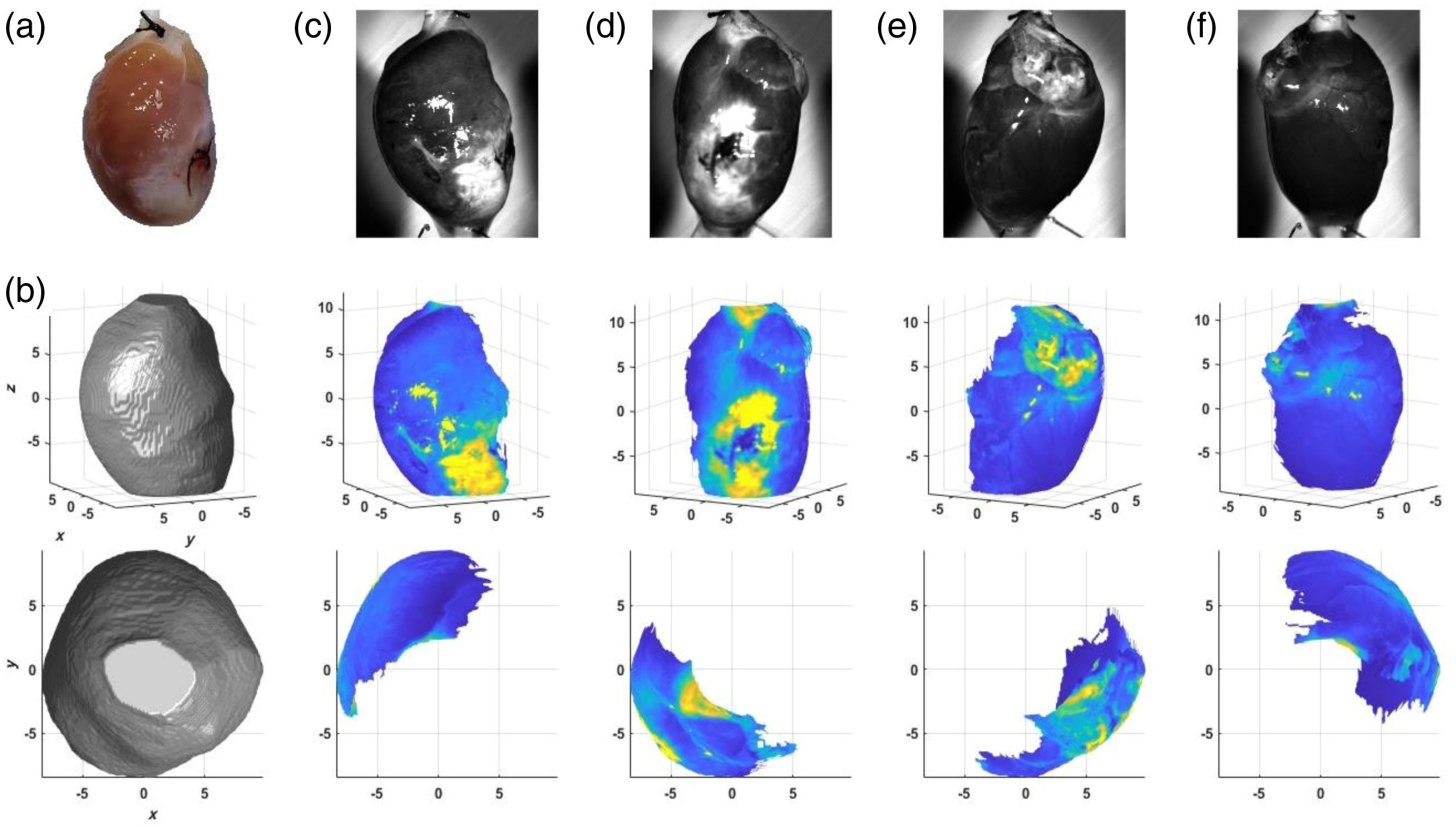
Heart geometry reconstruction with texture mapped tissue fluorescence that reveals collagen content across the entire epicardial surface. (a) RGB image of a heart at the end of an experiment showing the infarct and coronary ligature. (b) Side view and top view of the 3D model of the epicardial surface for the heart shown in panel (a). Units are mm. (c)–(f) Images of four viewpoints (90 deg intervals) of the heart shown in panel (a) during the epicardial geometry scan of the imaging protocol (Fig. 2). Top row: geometry images at each viewpoint showing fluorescence ($\lambda_{\mathrm{em}}=405\pm 20\;\mathrm{nm}$) that corresponds to connective tissue, primarily collagen. Middle row: side views of the 3D model (b) at each viewpoint that was texture mapped using each geometry image shown in the top row. Only the texture mapped viewpoint surface is shown for each viewpoint. Bottom row: top views of the 3D model (b) at each viewpoint that was texture mapped using each geometry image shown in the top row. Only the texture mapped viewpoint surface is shown for each viewpoint.

Collagen fluorescence, HSI spectral characteristics, normalized optical APs, and electrophysiological measurements (activation time and APDs) texture mapped onto the 3D epicardial mesh (Fig. 4) enabled quantitative correlative tissue structure-function analyses. Such data were assigned to mesh elements using 2D datasets obtained from each camera viewpoint, as previously described.^15^^,^^18^ Mesh elements within regions that were imaged by more than one camera view (overlapping regions) were assigned to only one view using an algorithm that connected elements along the midline of overlapping regions. Elements on either side of the midline were assigned to the corresponding camera view for texture mapping of data from that view. A textured model was then viewed using 3D rendering in MATLAB or as a 2D Hammer projection map.

#### 2.4.5 Domain-guided k-means clustering for structural and functional region classification

Data texture mapped to each cell in the 3D epicardial mesh were analyzed using a k-means clustering algorithm (Matlab k-means++ function “kmeans”)^42^^,^^54^ to identify regions with similar structural (tissue type) and functional (action potential morphology) characteristics. The algorithm used a heuristic approach to find centroids of ‘k’ distinct clusters. Beginning with a randomly chosen cell, the algorithm calculated the distance from the data value at that cell to the same value for every other cell in the mesh, and each cell was assigned to the closest cluster centroid. Through an iterative process, cells were assigned to a region to minimize the distance within clusters.

Before clustering, a histogram of the textured data was displayed for the user to select a threshold to remove specular reflection. Sites with data above that threshold were assigned values of NaN. A spatial 3×3 mean filter was then applied to reduce noise, which also reduced specular blooming due to the NaN values assigned to sites of specular reflection. All NaN values were then replaced with zeros, and the data were clustered using the kmeans function. The number of clusters for structural and functional region classification was determined using a domain-guided approach that was based upon physiology and prominent signal features.

The spectral integral from 400 to 435 nm corresponds mainly to collagen fluorescence (Table 2), a primary component of infarcted myocardium. This was therefore chosen to identify regions with similar structural characteristics. Clustering of spectral integral data resulted in five separate groups that were associated with (1) background and zero values, (2) healthy tissue, (3) border zone tissue, (4) infarcted tissue, and (5) saturated values. Sites having data within the background and zero value cluster and the saturated value cluster were removed from further analysis. We then examined if maps of the remaining three groups (healthy, border zone, and infarcted tissue) aligned with visual and histological assessments of the three types of tissue present within an infarcted ventricle.

Optically mapped action potentials were then clustered to identify regions with similar functional characteristics. Clustering resulted in groups of signals typically observed during optical mapping experiments: (1) those having motion artifact, (2) those having artifact due to dripping perfusate, and (3) action potentials without visible artifact. Action potentials for cells in the group without visible artifacts were then analyzed to determine if APDs were different between cells that were assigned to each of the three structural groups discussed above.

#### 2.4.6 Statistical analyses

Data are presented as mean ± standard deviation, and 95% confidence intervals were plotted to illustrate differences between groups, as noted in the figure captions. Figures were generated using MATLAB and GraphPad Prism.

## 3 Results

We designed and tested a multimodal high spatial resolution panoramic optical mapping system [Figs. 1(b) and 1(c)] that combines, across the entire epicardial surface of perfused hearts, tissue fluorescence spectra imaged during UV illumination with optically mapped action potentials, enabling electrophysiology to be analyzed with respect to local tissue type (Fig. 2). A rotational stage incrementally rotated hearts as images were acquired for the generation of a triangular mesh of the epicardial surface. At 90 deg increments, a translational stage also raised hearts in front of a line-scan hyperspectral camera to generate hyperspectral data cubes (30×30  mm, 448 wavelengths) to map epicardial tissue as healthy, infarct, or periinfarct (border zone). Transmembrane potential was optically mapped by imaging RH237 with four high speed CMOS cameras positioned around the heart every 90 deg. All cameras were registered to one coordinate frame using a custom calibration procedure (Fig. 3), enabling spectra and optical AP data to be assigned to each mesh element of the epicardial surface for correlative panoramic optical mapping.

### 3.1 Surface Reconstruction Accuracy and Resolution

Surface reconstruction accuracy was determined using images acquired from a geometry scan of a 19 mm diameter Delrin sphere (Table S1 in the Supplementary Material). For three subdivision levels of the initial octree,^15^ differences between the diameter and volume of the reconstructed surface of the sphere and the actual values were less than 2% and 4%, respectively. Surface discretization was more pronounced for the sixth subdivision, although the percent differences in diameter and volume were less (Fig. S1 in the Supplementary Material). Diameter and volume percent differences were similar at the seventh and eighth subdivision levels, but computation time increased to more than five hours for the eighth level. Considering these results, epicardial surfaces were reconstructed using seven subdivisions, with an initial 25 mm cubic bounding volume, to reduce both computation time and visible surface discretization. Reconstructed epicardial surface models had an average spacing between cell centroids of 0.104±0.021  mm.

### 3.2 Texture Mapping of Epicardial Data

Epicardial data texture mapped onto reconstructed surfaces provided detailed panoramic visualization that was seamless between imaging viewpoints. Texture mapped tissue fluorescence using images from geometry scans revealed regions with high collagen content, including the infarct scar, large vessels, and the atria (Fig. 4). The coronary ligature could also be identified. The co-location of features within geometry scan images and the corresponding texture mapped surface qualitatively confirmed that texture mapping was accurate and contiguous across a seam that joined images from two viewpoints (Fig. S2 in the Supplementary Material). Orienting the texture mapped surface with original images acquired at each viewpoint provided further visual confirmation of texture mapping accuracy [Figs. 4(c)–4(f)]. Side views and top views of surface regions that were texture mapped using each geometry scan image show the surface region imaged at each viewpoint. Comparing the top views identified surface regions imaged by more than one viewpoint, within which the overlapping region algorithm (methods section) determined which viewpoint image was assigned to each mesh cell in the overlapping region for texture mapping. A Hammer projection of texture mapped data across the entire surface provided two-dimensional maps of epicardial datasets and further confirmation of seamless texture mapping between viewpoints [Fig. 6(a)].

### 3.3 Hyperspectral Data Analysis

**Fig. 5:**
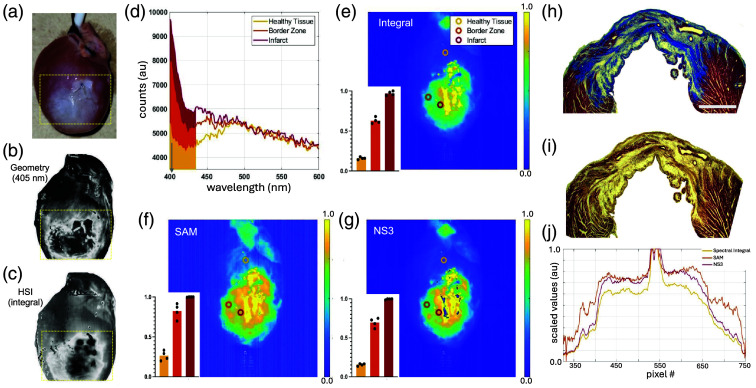
Hyperspectral panoramic mapping of a MI and spectral characteristics for local tissue type classification. (a) RGB image of a heart with an MI. The yellow rectangle denotes the same epicardial region in panels (a)–(c). (b) Tissue fluorescence ($\lambda_{\mathrm{ex}}=365+/-5.5\;\mathrm{nm}$, $\lambda_{\mathrm{em}}=405+/-20\;\mathrm{nm}$) imaged using the geometry camera and texture mapped onto the epicardial mesh generated for the heart shown in panel (a). (c) Integral of spectral intensity ($\lambda_{\mathrm{ex}}=365+/-5.5\;\mathrm{nm}$) from 400 to 435 nm computed using HSI data and texture mapped onto the epicardial mesh generated for the heart shown in panel (a). (d) Spectral intensities from 400 to 600 nm measured from a different heart at the three locations (circles) shown in panel (e) that reside within healthy, border zone, and infarcted tissue. The shaded regions indicate the spectral band (400 to 435 nm) over which intensities were integrated. The grey arrow at 405 nm indicates the emission wavelength of tissue fluorescence imaged using the geometry camera. (e)–(g) Images of normalized spectral characteristics computed using HSI data acquired from one heart. The insets show the average normalized characteristics of all hearts (n=4) for the 3 locations (circles) indicated on the images. (e) Integrated spectral intensity from 400 to 435 nm. (f) SAM [Eq. (1)]. (g) NS3 [Eq. (3)]. The infarct spectrum shown in panel (d) was used as the reference spectrum to compute SAM and NS3. (h) Histology image of a section of the LV infarct stained with Masson Trichrome, where blue indicates connective tissue and red indicates cytoplasm. (i) Histology image of the same of the LV infarct shown in panel (h) but stained with Movat Pentachrome, where black indicates nuclei and elastin, and yellow indicates collagen fibers and reticular fibers; red is muscle. (j) Normalized spectral characteristics (integral, SAM, and NS3) at each pixel along a line drawn across the epicardial region of the infarct.

Texture mapping of spectral characteristics measured from HSI data ($\lambda_{\mathrm{ex}}=365\pm 5.5\;\mathrm{nm}$, $\lambda_{\mathrm{em}}=400$ to 1000 nm) revealed regions of high collagen content across the epicardial surface, providing high contrast visualization of the infarct (Fig. 5). This result was qualitatively similar to that provided by the tissue fluorescence images of the geometry camera ($\lambda_{\mathrm{ex}}=365\pm 5.5\;\mathrm{nm}$, $\lambda_{\mathrm{em}}=405\pm 20\;\mathrm{nm}$), however, HSI spectral characteristics improved the delineation of borders between healthy, border zone, and infarcted tissue [Figs. 5(a)–5(c)]. Spectral differences between these three tissue types occurred between 400 and 475 nm, with infarcted tissue having the highest intensities [Fig. 5(d)]. Across all hearts, spectral intensity integral, SAM, and NS3 were significantly different for manually selected locations within healthy, border zone, and infarct core tissue locations [Figs. 5(e)–5(g)]. NADH fluorescence was low (Table 2) throughout each study due to maintenance of appropriate coronary flow and oxygenation and was therefore not analyzed.

Histological analysis of the LV confirmed that all infarcts were transmural and consisted of dense connective tissue. Connective tissue and nuclei were identified using Masson Trichrome stain [Fig. 5(h)], while Movat Pentachrome stain identified elastin and collagen [Fig. 5(i)]. The infarct of all hearts consisted primarily of collagen with only minor amounts of elastin observed within the border zone tissue. These histological results aligned with changes in HSI spectral characteristics across an infarct, with highest characteristic values corresponding to the infarct core and a transition to lower values that corresponded to the border zone [Fig. 5(j)]. Although SAM appeared to be more sensitive to spectral noise than spectral intensity integral and NS3, SAM is likely to be more useful in identifying differences in spectral morphology that could be generated by tissue regions having high protein heterogeneity. Spectral intensity integral and NS3 provided higher contrast between healthy, border zone, and infarcted tissue for a 4-week infarct, possibly due to the dense homogeneous expression of collagen, compared with elastin, within the infarct core and moderate collagen expression within border zone tissue.

**Fig. 6:**
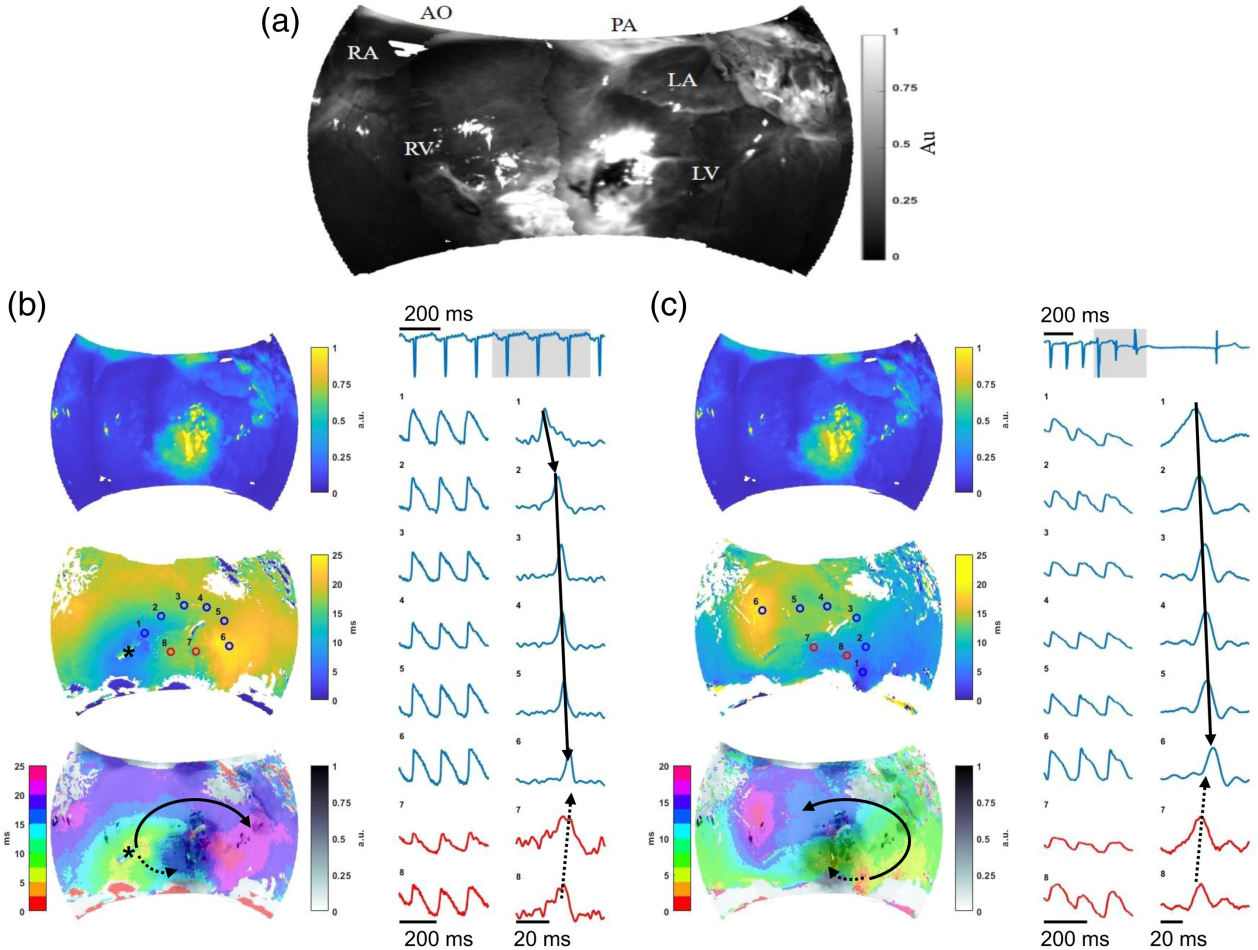
Correlative panoramic optical mapping of activation time and tissue spectral characteristics for paced and reentrant beats within an infarcted heart. (a) Hammer projection of panoramically mapped normalized tissue fluorescence ($\lambda_{\mathrm{ex}}=365\pm 5.5\;\mathrm{nm}$, $\lambda_{\mathrm{em}}=405\pm 20\;\mathrm{nm}$) using images acquired by the geometry camera for the heart shown in Figs. 5(e)–5(g). Major anatomic landmarks are annotated. (b), (c) A panoramically mapped paced beat (b) and a reentrant beat (c) for the heart shown in panel (a). The top image is a Hammer projection of the texture mapped normalized spectral intensity integrated from 400 to 435 nm. The middle image is a Hammer projection of texture mapped activation times computed for the second beat shown in the shaded region of the electrogram (top right) during pacing at a cycle length of 150 msec (b) or the first reentrant beat after a premature S2 (c). The bottom image is an overlay of the Hammer projections of the spectral integral and activation maps. Activation time is shown in color (scale on the left), and spectral integral is shown in greyscale (scale on the right). In panel (b), the pacing site is denoted with an asterisk. In panels (b) and (c), the lines indicate propagation direction, where the solid line denotes the main wavefront and the dotted line denotes a wavefront that encountered block. The right side of each panel in (b) and (c) shows an electrogram (top) that indicates global electrical activity and optical APs from eight epicardial locations (circles on the activation maps) next to the first derivative of the optical AP for the beat shown in the activation map, where the arrows indicate the progression of activation. Optical APs from healthy tissue are blue; optical APs from infarcted tissue are red.

### 3.4 Correlative Panoramic Mapping

**Fig. 7:**
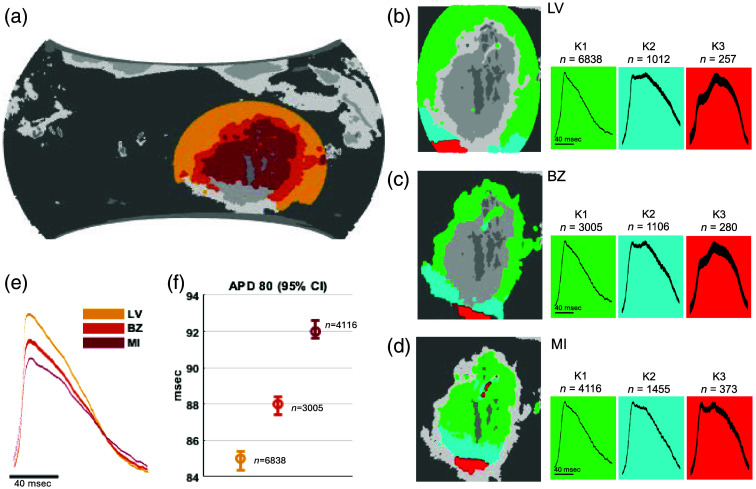
Structure/function correlative analysis of an infarcted heart using k-means clustering of spectral characteristics that correspond to tissue type and optical action potentials that correspond to electrophysiological function. (a) Hammer projection of tissue zones (LV, healthy; BZ, border zone; MI, infarcted) determined by k-means clustering of the spectral integral from 400 to 435 nm is shown in greyscale. Tissue zones within a circular region around the infarct are colored: healthy LV (yellow), BZ (orange), and MI (red). (b)–(d) Left: close-up maps of the k-means clustering of optical APs within each of the three tissue zones [healthy (top), border zone (middle), and infarcted (bottom)] shown in panel (a). Within each zone, three types of optical AP morphologies were identified by k-means clustering: K1 (green), K2 (cyan), and K3 (red). The 95% confidence interval of all APs within each type of morphology cluster is plotted in the colored boxes (right). The confidence interval for APs in group K1 was used to compute the APD80 95% confidence interval associated with each tissue zone for each animal. APs that were k-means clustered into groups K2 or K3 had either motion artifact (K2) or artifact caused by dripping perfusate (K3). (e) The 95% confidence interval for optical APs of the K1 group within each tissue zone shown in panel (a) are plotted together: healthy LV tissue (yellow), border zone (BZ) tissue (orange), and infarcted (MI) tissue (red). (f) Mean APD80 (circles) and 95% confidence interval (error bars) for the K1 group of each tissue zone shown in panel (a).

Texture mapping of tissue structure assessments (collagen fluorescence and HSI characteristics) and functional measurements (optical APs, activation time, and AP duration) to each cell within an epicardial mesh enabled quantitative correlative analyses of tissue type and electrophysiology (Figs. 6 and 7). Hammer projections of structural assessments and activation time identified areas of high collagen content, including the infarct, and mapped the movement of excitation waves across the epicardial surface (Fig. 6). Overlaying Hammer projections of spectral intensity integral with activation time (one example of correlative mapping) during paced beats revealed that excitation waves quickly propagated around the infarct and slowly propagated and blocked within the infarct [Fig. 6(b), left]. Compared with healthy tissue, optical APs within infarcted tissue had lower signal-to-noise ratios and longer durations [Fig. 6(b), right]. Correlative mapping also revealed the origin and propagation of ectopic beats. For example, correlative mapping of an ectopic beat after a premature S2 showed that the early site of activation was within the apical infarct border zone, and the wave slowly propagated through the infarct while quickly propagating around the infarct [Fig. 6(c)].

In addition to seamless quantitative analysis of AP propagation, correlative mapping also enabled APs to be selectively analyzed within specific tissue types. An illustrative demonstration of this for one heart revealed AP differences between healthy, border zone, and infarcted tissue (Fig. 7). This was done using a k-means algorithm to assign one of the three tissue types to cells of the epicardial mesh using each cell’s spectral intensity integral. Hammer projections of the assigned tissue type for a circular region around the infarct revealed distinct zones that corresponded to each of the three types of tissue, clearly demarking border zone tissue and the infarct core [Fig. 7(a)]. Further k-means sorting of the cells within each tissue zone using each cell’s optical AP revealed contiguous regions with similar AP morphologies, with the largest within-tissue-region associated with optical APs without artifact and smaller within-tissue-regions located at the apex that were associated with motion artifact and the dripping of perfusate [Figs. 7(b)–7(d), left]. AP dispersion for cells of each within-tissue-region was examined by plotting the 95% confidence interval for all the APs within the region, where the width of the 95% confidence interval corresponds to AP dispersion [Figs. 7(b)–7(d), right]. AP dispersion was highest for within-tissue-regions that were associated with artifacts. The 80% duration of the 95% confidence intervals (analogous to the confidence interval for 80% APD) for within-tissue-region APs that had no artifact were longest for infarcted tissue, shorter for border zone tissue, and shortest for healthy tissue [Fig. 7(e)]. This preliminary result was consistent for all four infarcted hearts that were analyzed and in line with prior studies showing prolonged APD in the failing myocardium.^44^^,^^55^^,^^56^

## 4 Discussion and Conclusion

There is long-standing interest in structure-function relationships as drivers of cardiac arrhythmias. Most life-threatening arrhythmias occur in hearts with altered structure and molecular composition, such as aging hearts with increased fibrosis and injured post-MI hearts.^29^^,^^30^^,^^57^ Many anti-arrhythmic therapies target specific structural sites, such as the pulmonary veins for treating atrial fibrillation.^58^ Understanding these structural and molecular correlates of arrhythmias will improve arrhythmia therapies. Previous work has pursued this goal by combining multiple imaging modalities to study living hearts, followed by post-fixation imaging.^22^^,^^59^^,^^60^ This has provided new insight into fundamental processes, including cardiac pace-making and pathological activation of injured hearts, including the human heart. In computational clinical cardiology, the structure-function relationship has a key role in predicting arrhythmias in patients^32^^,^^61^^,^^62^ in a personalized “digital twin” manner. This has fueled innovations in clinically relevant imaging modalities and their integration with computational modeling.^63^

Our study is a noteworthy contribution to the arsenal of tools to investigate cardiac structure-function relationships in the *ex vivo* heart to better understand arrhythmia mechanisms. We present the first all-optical panoramic imaging system for living hearts that combines 3D geometry imaging, optical mapping of action potentials, local tissue characterization using HSI, and a quantitative approach that fuses those panoramic datasets. We demonstrate how to reliably reconstruct the 3D geometry of the heart and how to texture-map various aspects of molecular composition, metabolic state, and electrical function onto that 3D geometry. Using a healed MI rat model, we have demonstrated that paced activity yields distorted wave trajectories due to MI tissue remodeling (collagen and elastin deposition), identified by spectral analysis, and that the identified border zone is a frequent site of extra beats (Fig. 6), as predicted due to cells having a more depolarized state. Importantly, we provide an example of how automated analysis of the HSI datasets yields physiologically relevant regions (healthy, border zone, and MI) that indeed have unique electrophysiological signatures based on the mapped optical action potentials (Fig. 7). To our knowledge, this is the first seamless fusion of these imaging modalities that has been applied across the surface of the heart.

In future developments, the time required for panoramic imaging could be reduced by replacing the line-scan HSI camera with an area-scan camera to avoid the vertical scans of the heart. Further speedup in geometry reconstruction could be achieved by pursuing newer methods, such as computationally enhanced foveal ghost imaging using curved mirrors^64^ and single-shot acquisition through two or more cameras. The spatial resolution could be increased using many low-cost machine vision cameras,^27^^,^^65^ strategically positioned to improve apical and basal imaging. This would allow for more detailed co-registration of perturbed wavefronts with molecular and structural alterations. Glare artifacts from dripping perfusate could be eliminated by submerging the heart in a bath and optimizing the illumination.

Other limitations of the current approach include the need to inhibit contraction and the inability to image transmurally to the endocardial surface. Panoramic imaging of electrical activity in contracting hearts^25^^,^^66^ is possible but requires sophisticated computational methods to reliably separate signal components that correspond to electrical activity and contraction. Doing that for all modalities applied here, including the HSI, is possible with further considerations, such as tagged acquisition of spectra during the cardiac cycle. Faster acquisition of geometry and of spectra would support this goal. Being able to perform both electrical mapping and HSI in a working heart^35^ is essential for studies of regional metabolic changes that may affect electrical activity.

Previously, panoramic endocardial mapping of electrical activity in sheep atria was done using ultra-wide view lenses and two cameras.^67^ This is not feasible for smaller rodent hearts. Transmural imaging of any of the parameters of interest: structure, molecular composition, and electrical activity, is of great value yet difficult to do panoramically. For transmural optical mapping of action potentials, Pertsov and colleagues developed several methods that combine trans- and epi-illumination with computational reconstruction of photon migration.^68^^–^^71^ Some of these concepts are applicable to our panoramic system, not only for activation mapping but also for tissue spectral analysis. In rabbit heart studies that measured changes in myocardial absorbance to analyze mitochondrial redox state, the light source was positioned inside the ventricles for transillumination while spectra were acquired from the epicardium.^36^^,^^72^ This transillumination approach is also possible for rodent hearts and could be incorporated into our system.

The most impactful future advances of the presented imaging approach are likely to come from further development of the HSI data analysis. For example, scanning multiple excitation and emission frequencies to acquire the full excitation-emission data matrix would provide a finer portrait of the molecular composition and any changes within myocardial tissue. Spectral unmixing using known spectra for extracellular matrix proteins, as done previously for myoglobin, cytochrome-C, and NADH,^72^ could be applied, especially for collagen and elastin.^73^ This would enable a quantitative analysis of molecular composition at each spatial location. Furthermore, it could be possible to map fiber orientation using HSI data if the illumination light were polarized.^74^ Fiber anisotropy could be measured using this approach to provide an important structural characteristic that determines activation wave speed and morphology.^75^

The all-optical panoramic imaging of structure and function presented here is a powerful tool to investigate a variety of tissue features that impact cardiac arrhythmias. Structural abnormalities can be induced by MI, as shown here, or by heart failure that promotes diffuse fibrosis, a condition that could be measured using HSI. Aging-related fibrosis and changes in heart composition, including increased fat deposition, also provide a substrate for cardiac arrhythmias that could be measured by the presented system. Other applications may include investigations of engraftment arrhythmias and tissue remodeling after stem cell therapy.^76^ An important area where the presented system would be a powerful investigative tool for treating arrhythmias is the placement of ablation lesions to block abnormal conductive pathways using various energy modalities, including PFA. It is possible that different ablation modalities generate different lesion spectral signatures, and the current system could be used to investigate associated changes in tissue composition and electrical activity. Abnormal conduction pathways can also be blocked using optogenetics. Optogenetic “ablation” could be studied using the presented panoramic system alongside optical actuation of light-sensitive proteins, where light patterns could be projected onto the tissue to temporary block activation, providing a valuable panoramic approach to test and optimize the positioning of anti-arrhythmic lesions.^77^^,^^78^

In summary, we developed a new multimodal panoramic system that integrates mapping of electrical activity and spectral analysis of tissue composition across the surface of living hearts. This imaging approach will fuel new scientific investigations and insights for the prevention and treatment of cardiac arrhythmias.

## Supplementary Material

10.1117/1.JBO.31.7.076004.s01

## Data Availability

The code and data presented in this article are publicly available in FigShare at https://figshare.com/articles/software/Code_and_data_for_hyperspectral_panoramic_optical_mapping_project/31129714.
