# Supplementary material for: Panoramic hyperspectral optical mapping of cardiac membrane potential and tissue type
Source: J Biomed Opt. 2026 Jul 22;31(7):076004. doi: 10.1117/1.JBO.31.7.076004 (PMC13391206; doi:10.1117/1.JBO.31.7.076004)
Supplement: Supplementary file 1 [file JBO_031_076004_SD001.pdf]

Panoramic hyperspectral optical mapping of cardiac membrane potential and tissue type

Grant Kowalik[1], Rebekah Russo[1], Murray Loew[1], David Mendelowitz[2], Emilia Entcheva[1], Matthew W. Kay[1]

[1]Department of Biomedical Engineering, School of Engineering and Applied Science, The George Washington University, Washington, District of Columbia.

[2]Department of Pharmacology & Physiology, School of Medicine and Health Sciences, The George Washington University, Washington, District of Columbia.

Address for Correspondence:

Matthew W. Kay, DSc
Professor of Biomedical Engineering
School of Engineering and Applied Science
The George Washington University
Suite 5000 Science and Engineering Hall
800 22nd St NW,
Washington, DC 20052
Tel: 202-994-2898
Email: phymwk@gwu.edu

Supplemental data

Sub-divisions	Trimesh faces	Voxel edge length (mm)	Computation time (min)	Reconstructed dia (mm), % error	Reconstructed vol (mm^3), % error
6	22492	0.195	0.64	19.141, 0.74%	3724, 3.71%
7	90128	0.098	14.35	19.336, 1.77%	3730, 3.88%
8	366028	0.049	336.28	19.238, 1.25%	3730, 3.74%

Supplemental Table 1: Geometry reconstruction results for a smooth sphere. A 19mm diameter (3590 mm^3 vol) sphere was scanned to measure the accuracy of surface reconstruction. The surface was reconstructed using an initial bounding volume of 25 mm^3 consisting of 8 voxels with volumes of 12.5 mm^3. The surface geometry was reconstructed after the initial voxels were subdivided 6, 7, and 8 times using an adaptive octree mesh refinement algorithm. The diameter and volume of the reconstructed sphere at each final subdivision is shown in columns 5 and 6. Camera resolution at object distance was 0.333 mm/pixel.

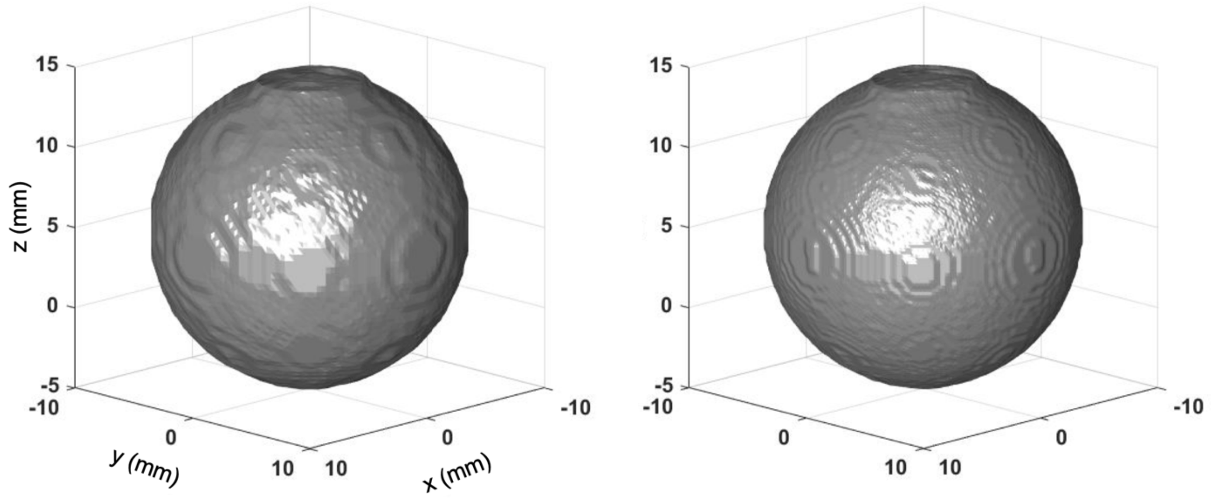

Supplemental Figure 1: Surface reconstructions of a 19mm diameter sphere using an initial bounding volume of 25x25x25mm. Surface resolution was dependent upon the number of subdivisions of the bounding volume. **Left**: Sphere surface for 6 subdivisions. **Right**: Sphere surface for 7 subdivisions. Seven subdivisions were used for all panoramic mapping of perfused rat hearts.

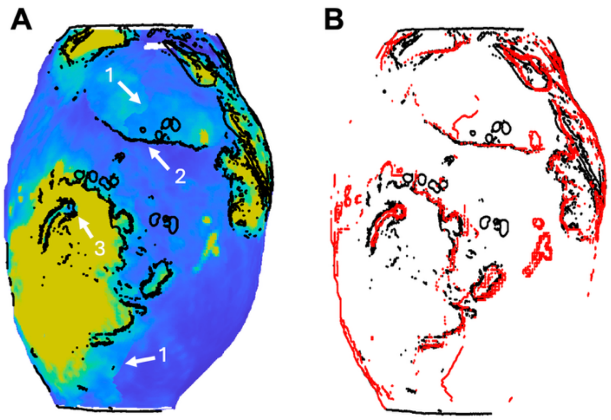

Supplemental Figure 2: Confirmation of accurate texture mapping. Features within geometry camera images and the corresponding texture mapped surface were co-located and contiguous across a seam that joined images from two viewpoints. **A**: Edge features from an original geometry scan image (black dots) are plotted on top of an image of the texture mapped surface that is oriented to the same viewpoint as the original geometry scan image. The black dots align with the edges of features within the texture mapped surface. “1” denotes a seam between images of two viewpoints. “2” denotes the edge of the left atrium, where the edge of the left atrium in the texture mapped surface aligns with the edge of the left atrium detected within the original geometry image (black dots). “3” denotes the location of the LAD suture, where the suture in the texture mapped surface aligns with the edge of the suture detected within the original geometry image (black dots). **B**: Edge features from an original geometry scan image (black dots) are plotted with edge features of the image of the texture mapped surface oriented to the same viewpoint (red dots). The edge features of both images are co-located.
